# Supplementary material for: Topic modeling identifies novel genetic loci associated with multimorbidities in UK Biobank
Source: Cell Genom. 2023 Aug 1;3(8):100371. doi: 10.1016/j.xgen.2023.100371 (PMC10435382; doi:10.1016/j.xgen.2023.100371)
Supplement: Document S1, Figures S1–S13 [file mmc1.pdf]

Cell Genomics, Volume *3*

Supplemental information

Topic modeling identifies novel genetic loci associated with multimorbidities in UK Biobank

Yidong Zhang, Xilin Jiang, Alexander J. Mentzer, Gil McVean, and Gerton Lunter

Supplemental Figures

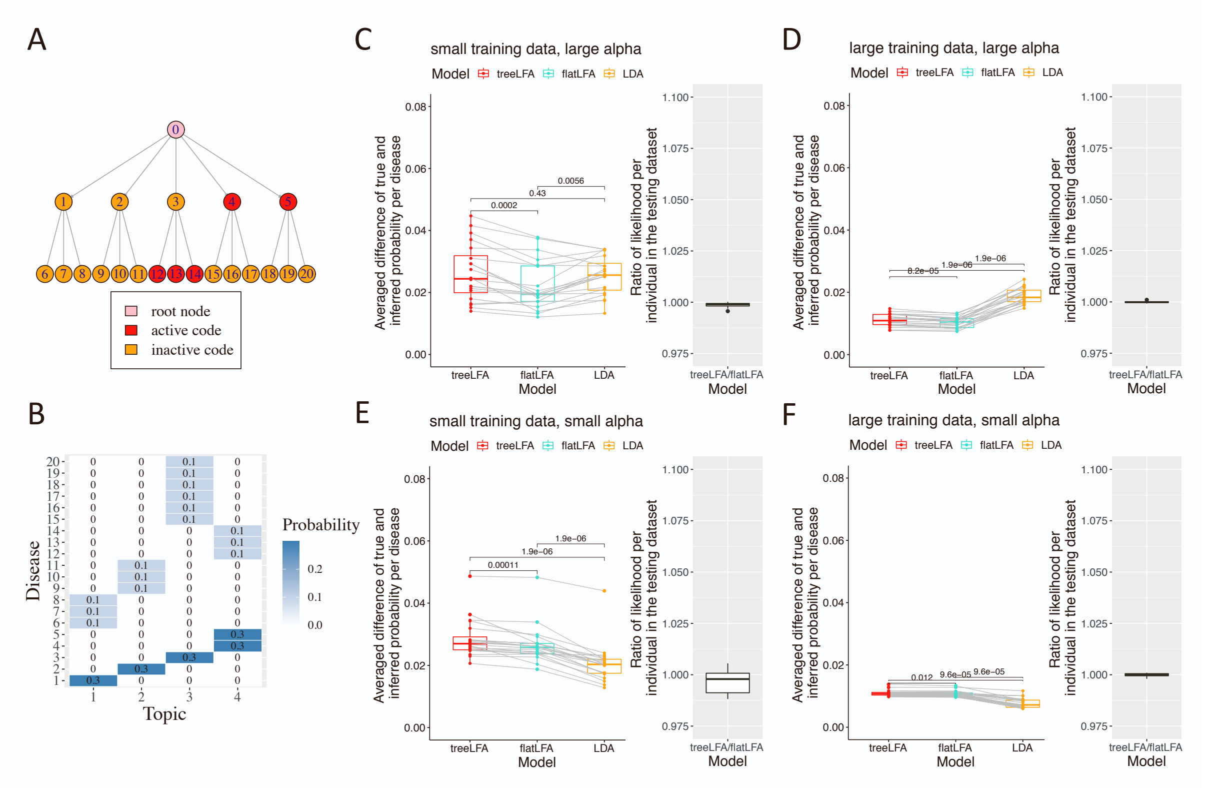

Supplemental Figure 1: Comparison of three related topic models (treeLFA, flatLFA and LDA) on simulated datasets, related to Figure 2. A.) The tree structure of 20 diseases. Red nodes correspond to the active codes in Topic 4 in panel B. **B.)** The four topics used for simulation. Active codes in these topics are unlikely to be generated by a Markov process with small probability of transforming from inactive to active and large probability of staying active while going from the parent node to its children nodes, since an active parent code always has inactive children codes, while active children codes always have inactive parent code on the tree structure in panel A. **C-F.)** Comparison of three topic models on simulated datasets. The Parameter setting and metrics are the same as those in Figure 2. **C.)** Results on datasets simulated using D=2500 and α=1. **D.)** D=5000 and α=1. **E.)** D=300 and α=0.1. **F.)** D=1000 and α=0.1. The numeric results are in Table S1B. Two sided paired Wilcox test (n=20) is used to compare inference accuracy between treeLFA and flatLFA/LDA.

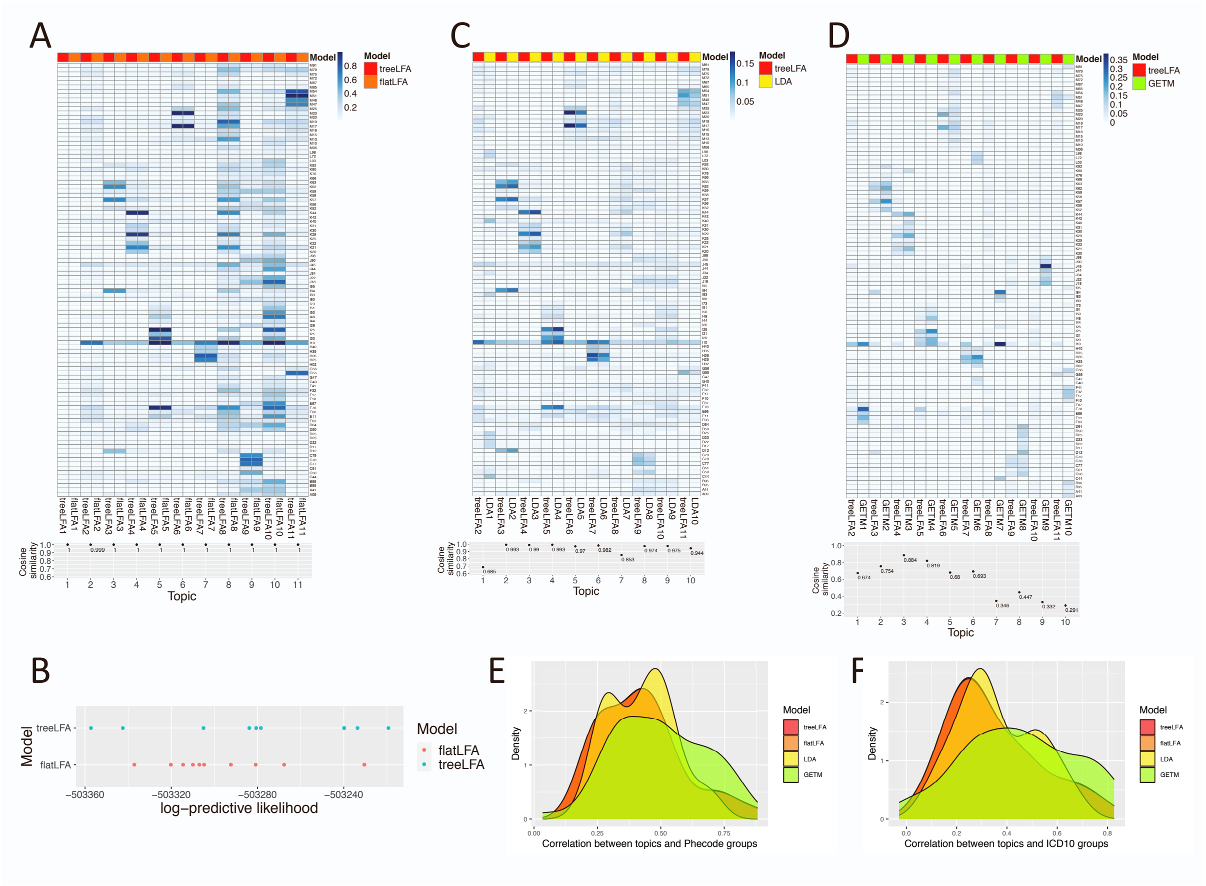

Supplemental Figure 2: Comparison of topics inferred by four topic models on the top-100 UKB dataset, related to Figure 3. A.) Comparison of the 11 topics inferred by treeLFA and flatLFA. The same topics inferred by the two models are placed next to each other. Cosine similarity was used to measure the similarity of topics inferred by the two models (point plot below the heatmap). The numeric results are in Tables S2B, S3A. **B.)** The log10-predictive likelihood of the ten treeLFA and flatLFA chains on the test data. For each chain, topic vectors averaged from the 50 posterior samples of topics are used to calculate the predictive likelihood. Note that the predictive likelihood of treeLFA and LDA based topic models cannot be compared directly, as treeLFA includes the number of diseases as a feature in the model, whereas LDA conditions on this number. Instead, we focused the comparison on genetic association studies (see the "GWAS on topic weights" subsection). **C-D.)** Comparison of the 10 topics inferred by LDA (panel C)/GETM (graph-embedded topic model, panel D) and the 10 non-empty topics inferred by treeLFA. LDA/GETM models with 10 topics were trained, as no empty topic would be inferred by LDA based models, so they were trained with one fewer topic than treeLFA. Topics inferred by treeLFA are normalised such that probabilities of the 100 ICD-10 codes add up to 1 in any topic. The numeric results are in Tables S2B, S3A. **E-F.)** The distribution of correlation between inferred topics and all Phecodes (panel E)/ICD-10 (panel F) groups for the four topic models on the top-100 UKB dataset. Only the top-5 largest correlations for each topic were kept and plotted. The numeric results are in Table S3C. Note that GETM had larger correlation with ICD10/Phecode chapters/categories than the other three models, possibly reflecting its use of strong priors for disease groupings.

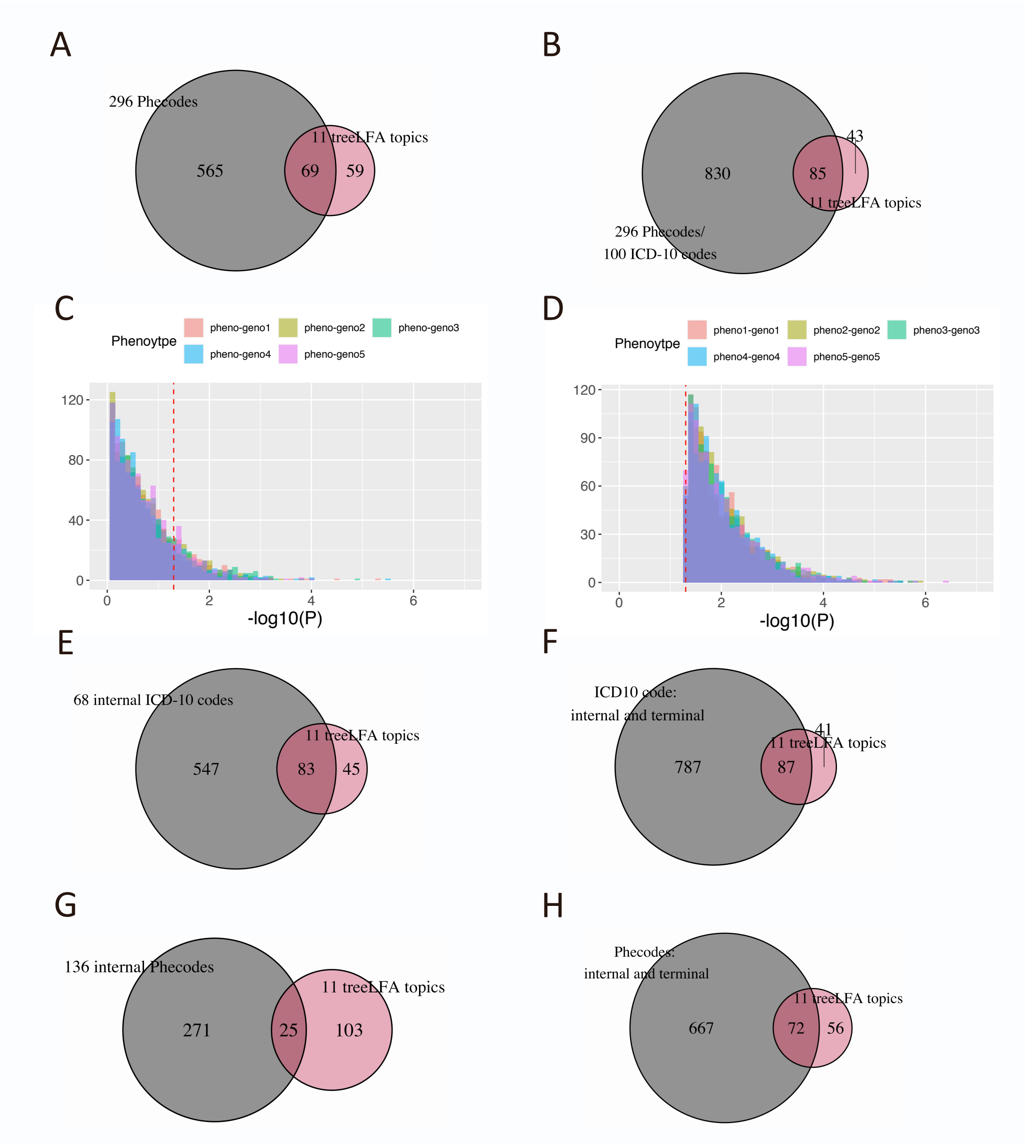

Supplemental Figure 3: Overlap of significant loci found by GWAS for different traits, related to Figure 4. A.) The total numbers of loci associated with any of the 296 Phecodes mapped from the 100 ICD-10 codes and any of the 11 treeLFA topics, and their overlap. **B.)** The total numbers of loci associated with any of the 296 Phecodes or the 100 ICD-10 codes and any of the 11 treeLFA topics, and their overlap. **C.)** P-values of the association between five simulated diseases and their corresponding associated SNPs. The simulation was repeated 1,000 times. **D.)** P-values of the association between the five SNPs in panel C and the binary phenotype combined from the five diseases in panel C. The simulation was repeated 1,000 times. **E.)** The total numbers of loci associated with any of the 68 internal ICD-10 codes and any of the 11 treeLFA topics, and their overlap. **F.)** The total numbers of loci associated with any of the 136 internal Phecodes and any of the 11 treeLFA topics, and their overlap. **G.)** The total numbers of loci associated with any of the internal or terminal ICD-10 codes and any of the 11 treeLFA topics, and their overlap. **H.)** The total numbers of loci associated with any of the internal or terminal Phecodes and any of the 11 treeLFA topics, and their overlap.

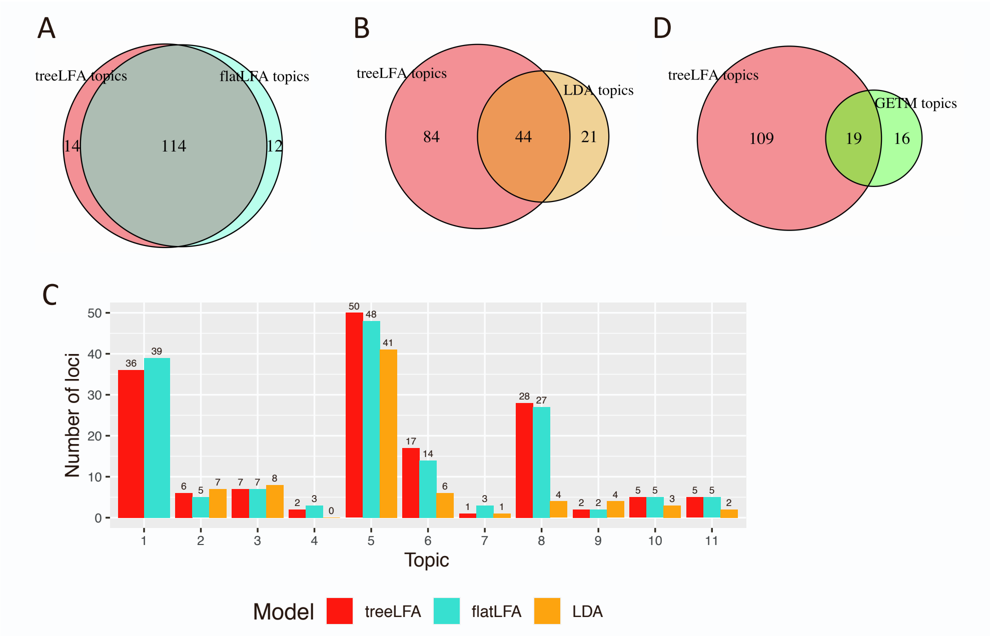

Supplemental Figure 4: Comparison of the topic-GWAS results for the topic models, related to Figure 4. A.) The total numbers of loci associated with any of the 11 topics inferred by treeLFA and flatLFA, and their overlap. **B.)** The total numbers of loci associated with any of the 11 topics inferred by treeLFA and any of the 10 topics inferred by LDA, and their overlap. **C.)** The numbers of loci associated with each of the 11 topics inferred by treeLFA and flatLFA, and each of the 10 topics inferred by LDA. The first topic is the empty topic, which is only inferred by treeLFA and flatLFA. **D.)** The total numbers of loci associated with any of the 11 topics inferred by treeLFA and any of the 10 topics inferred by GETM, and their overlap.

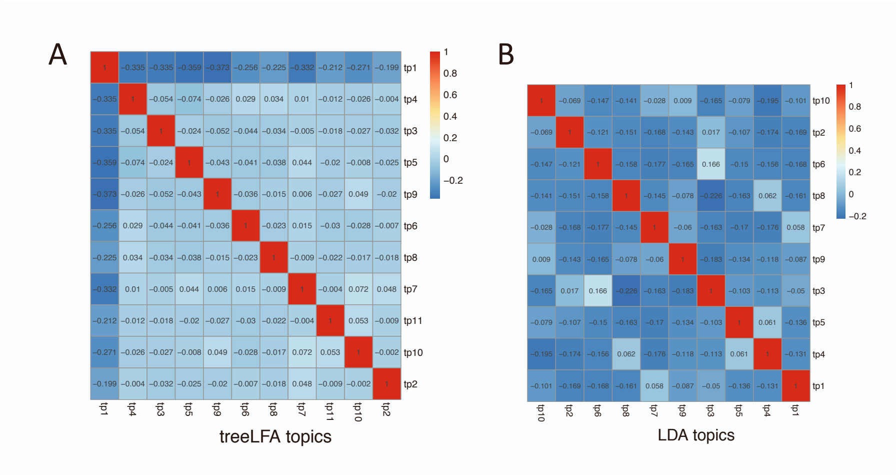

Supplemental Figure 5: Correlation of topic weights for topics inferred by treeLFA and LDA, related to Figure 4. A.) The correlation matrix for individuals' weights for the 11 topics inferred by treeLFA. The first topic is the empty topic. **B.)** The correlation matrix for individuals' weights for the 10 topics inferred by LDA. The matrix uses the same colour scheme as the matrix in panel A.

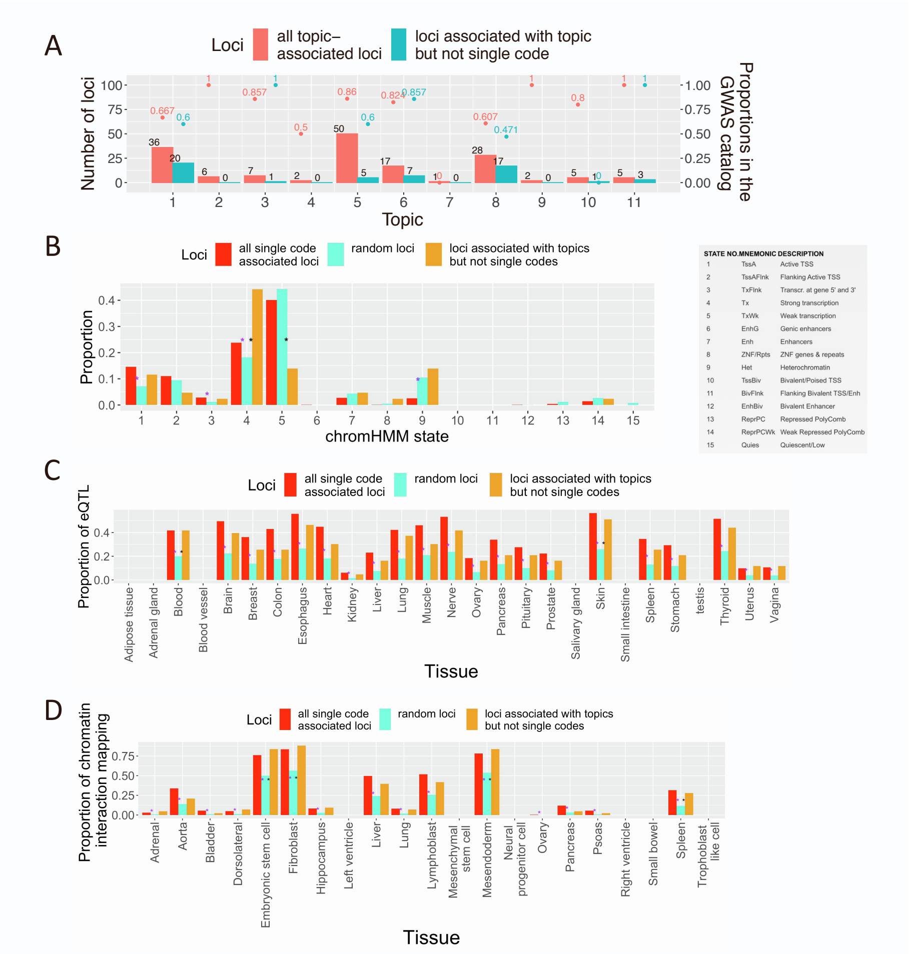

Supplemental Figure 6: **Validation of topic associated loci, related to Figure 4.** **A.)** The proportions of loci associated with the 11 treeLFA topics that are recorded in the GWAS catalogue. Red bars show the results for all topic-associated loci for each topic, and green bars show the results for loci that are associated with each topic but not any single code. **B.)** The proportions of three groups of SNPs that are in different chromHMM states. Meanings of different chromHMM states are shown in the right table. The first group contains lead SNPs associated with at least one ICD-10 code (698 lead SNPs); the second group contains 5,000 randomly selected SNPs (4,649 lead SNPs after clumping); The third group contains lead SNPs associated with at least one of the 11 treeLFA topics but not any single code (43 lead SNPs). The proportions of the first and third groups are compared with the second group respectively, and significant differences in proportions (two sided two-proportion Z-test, P-value<0.05, Bonferroni correction for the 15 chromHMM states) are marked with asterisks between the corresponding bars (purple asterisks mean significant differences between the first and second groups, black asterisks mean significant differences between the third and second groups). The numeric results are in Table S5B. **C.)** Proportions of the three groups of SNPs in panel B being eQTL in different tissues. Two sided two-proportion Z-test, P-value<0.05, Bonferroni correction for the 27 tissues. The numeric results are in Table S5B. **D.)** Proportions of the three groups of SNPs in panel B having chromatin interaction with genes in different tissues. Two sided two-proportion Z-test, P-value<0.05, Bonferroni correction for the 21 tissues. The numeric results are in Table S5B.

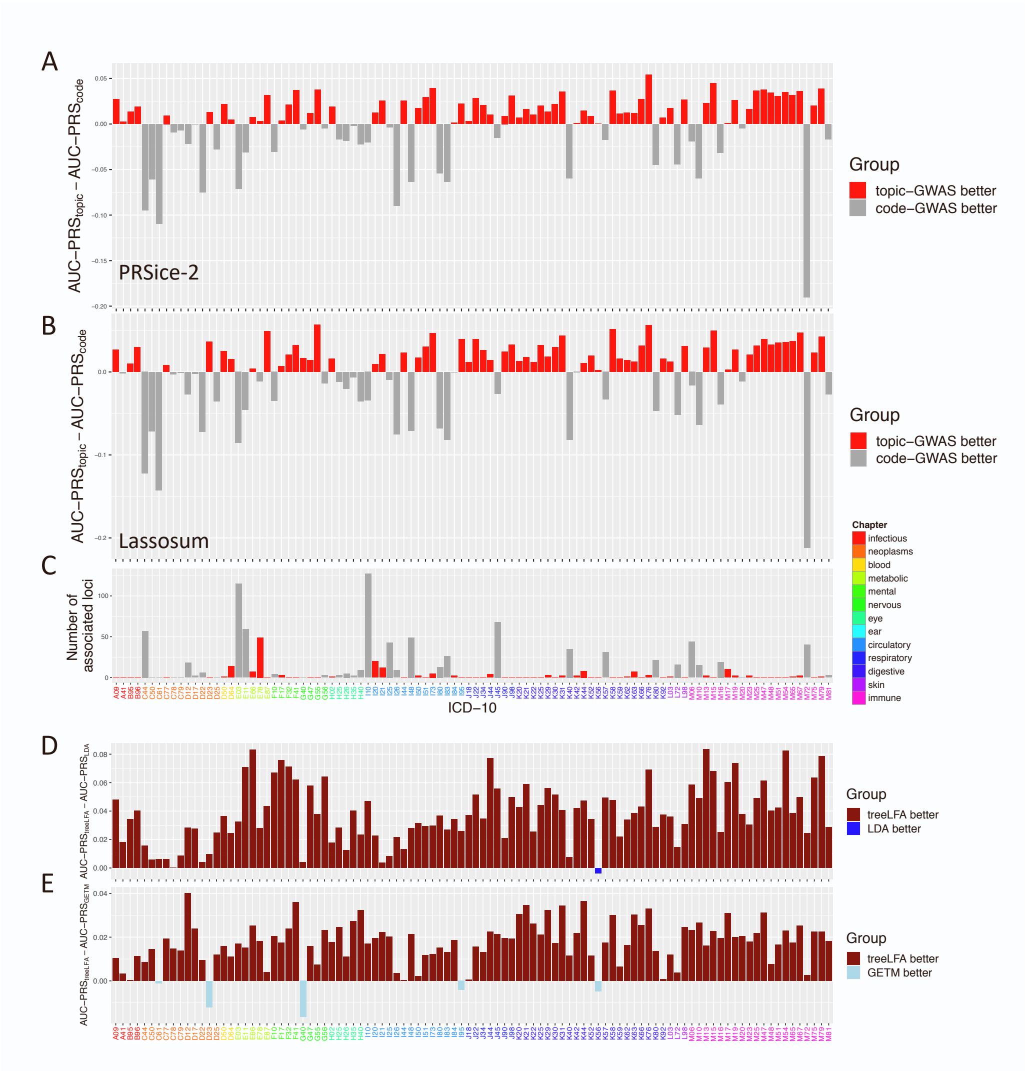

Supplemental Figure 7: **PRS for ICD-10 codes based on topic-GWAS results, related to Figure 4. A.)** Comparison of the AUC of two types of PRS for the 100 ICD-10 codes on the test data. One type of PRS is constructed using topic-GWAS results for treeLFA, and the other type of PRS is constructed using single code GWAS results. Bars are coloured according to the relative performance of the two types of PRS. PRS are calculated using the software PRSice-2. The numeric results are in Table S5D. **B.)** Comparison of the AUC of the two types of PRS in panel A for the 100 ICD-10 codes on the test data. PRS are calculated using the software Lassosum. The numeric results are in Table S5D. **C.)** The numbers of loci associated with each of the 100 ICD-10 codes. Bars are coloured the same way as in panel A. Codes are coloured according to the ICD-10 chapters they belong to. **D-E.)** Comparison of the AUC of PRS constructed using the topic-GWAS results for treeLFA and LDA (panel D)/GETM (panel E). Bars are coloured according to the relative performance of each pair of PRS. The numeric results are in Table S5E.

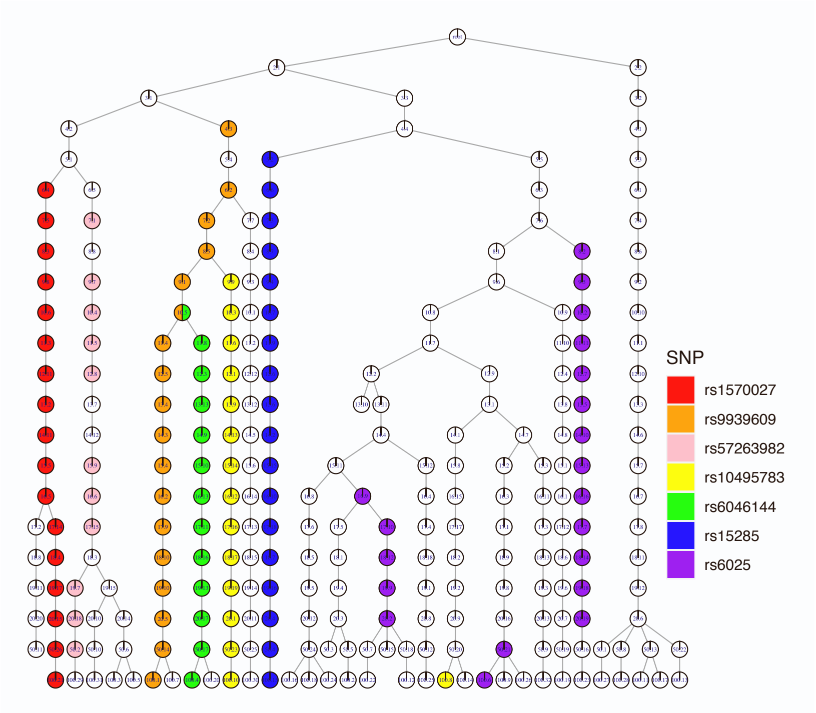

Supplemental Figure 8: Association of example SNPs and topics from different treeLFA models, related to Figure 5. The association of a few example SNPs and topics inferred by different treeLFA models are visualised on the tree structure of topics. The tree structure of topics is the same as the one in Figure 5B. Topics significantly associated with 7 different SNPs are highlighted with different colours on the tree structure. The numeric results are in Table S6B.

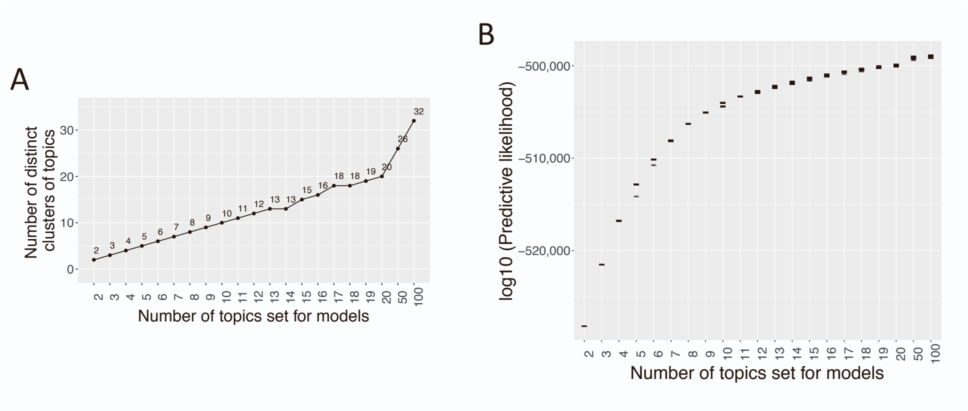

Supplemental Figure 9: Summary statistics for models with different numbers of topics, related to Figure 5. A.) Numbers of distinct topics remained after clustering for treeLFA models set with different numbers of topics. **B.)** The log10-predictive likelihood on the test data for the ten Gibbs chains for treeLFA models set with different numbers of topics. For each chain, the standard deviation of the predictive likelihood calculated using different posterior samples of topics from the chain are shown. The numeric results are in Table S6D.

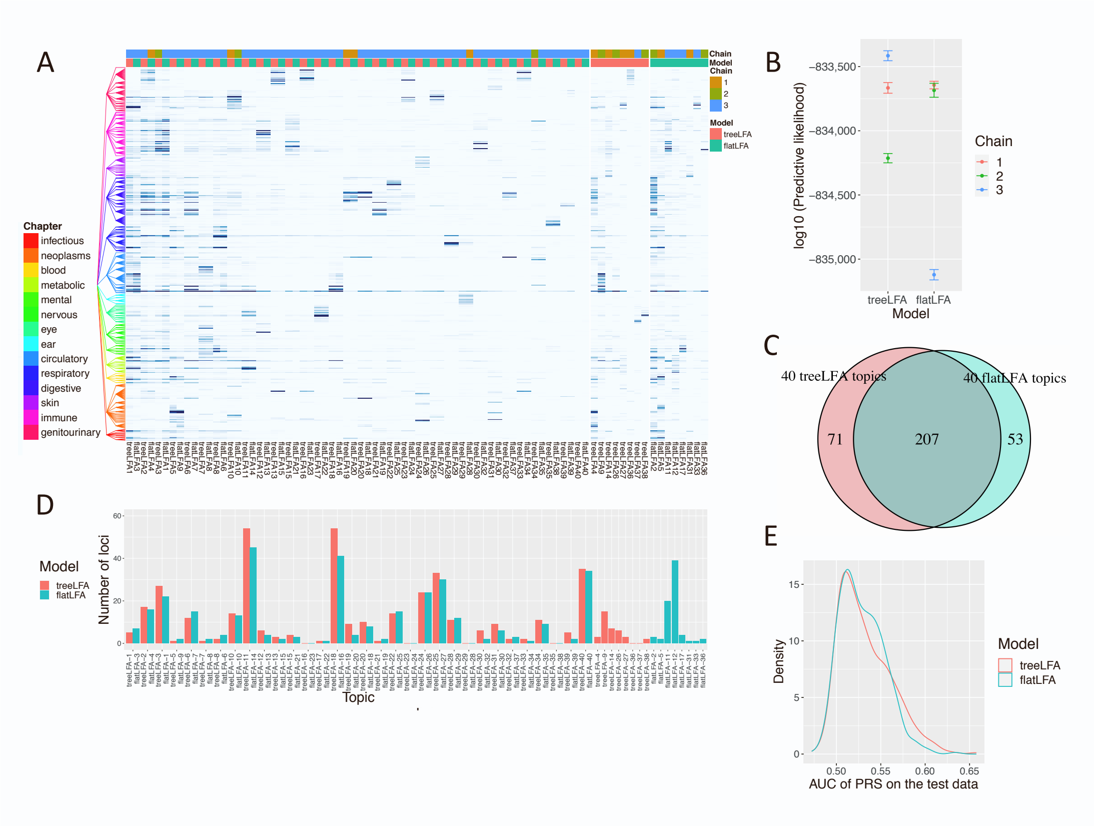

Supplemental Figure 10: Comparison of the inference and topic-GWAS results for treeLFA and flatLFA on the top-436 UKB dataset, related to Figure 6. A.) The 40 distinct topics inferred by treeLFA and flatLFA models set with 100 topics. The same topics inferred by treeLFA and flatLFA are placed next to each other. Topics inferred by both models are shown first, followed by topics inferred by only one model. For each model, the inferred topics are numbered according to their density. The tree structure of the 436 ICD-10 codes is shown to the left of the heatmap, and codes from different ICD-10 chapters are coloured differently. For each topic, the number of Gibbs chains that inferred it is shown with the colour bar on top of the heatmap. The numeric results are in Table S7B. **B.)** The log10-predictive likelihood on the test data for the three treeLFA and flatLFA chains. The calculation of predictive likelihood was repeated ten times for each chain to get the standard deviation. The numeric results are in Table S7D. **C.)** The total numbers of loci associated with any of the topics inferred by treeLFA and flatLFA, and their overlap. **D.)** The numbers of loci associated with each of the treeLFA and flatLDA topics. Topics have the same order as those in panel A. The numeric results are in Table S7F. **E.)** Density plots for the AUC of PRS for the 436 ICD-10 codes on the test data based on the topic-GWAS results for treeLFA and flatLFA. The numeric results are in Table S7K.

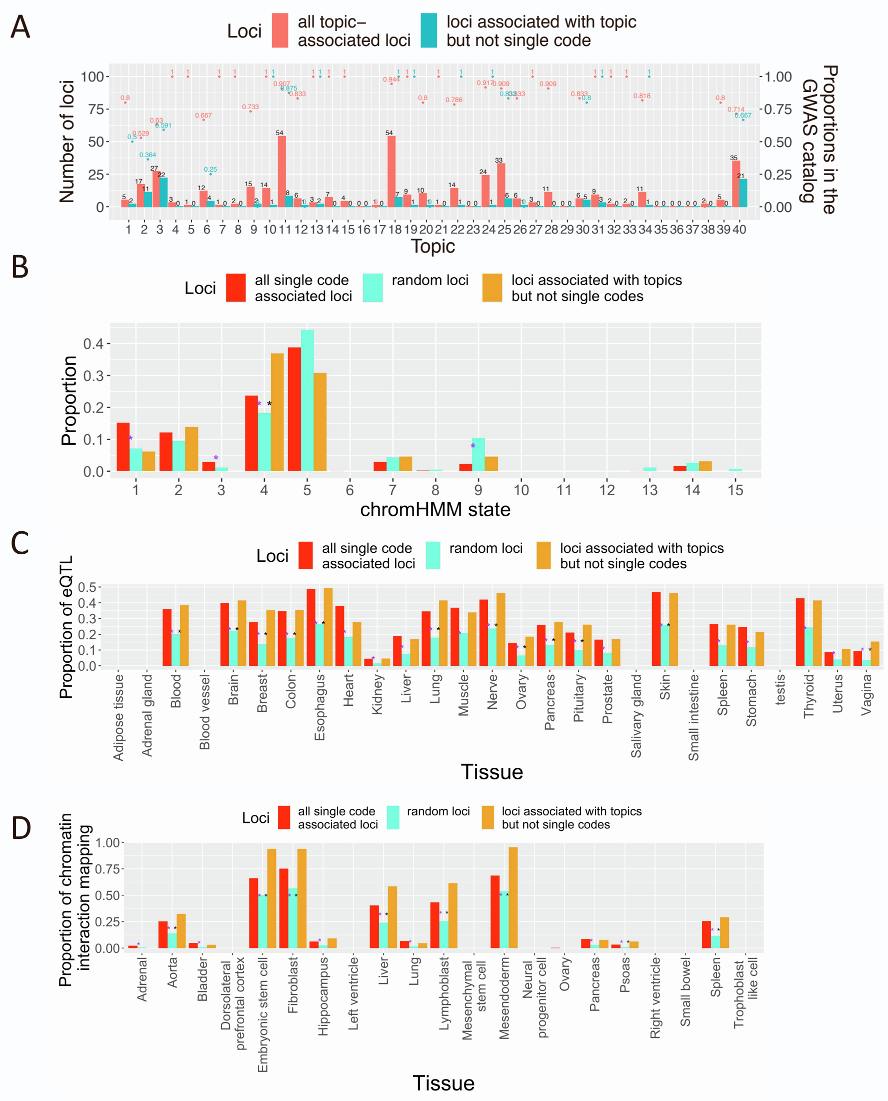

Supplemental Figure 11: Validation of topic associated loci on the top-436 UKB dataset, related to Figure 6. All settings are same as those in Figure S6. **A.)** The proportions of topic-associated loci recorded in the GWAS catalogue. Overall, 89.2% (248/278) of topic-associated loci and 78.9% (63/80) of unique associations have records in the GWAS catalogue. **B.)** Proportions of the three groups of SNPs (823 single code associated lead SNPs; 4,649 random lead SNPs; 65 topic-associated lead SNPs not found by single code GWAS) that are in different chromHMM states. The numeric results are in Table S7J. **C.)** Proportions of the three groups of SNPs in panel B being eQTL in different tissues. The numeric results are in Table S7J. **D.)** Proportions of the three groups of SNPs in panel B having chromatin interaction with genes in different tissues. The numeric results are in Table S7J.

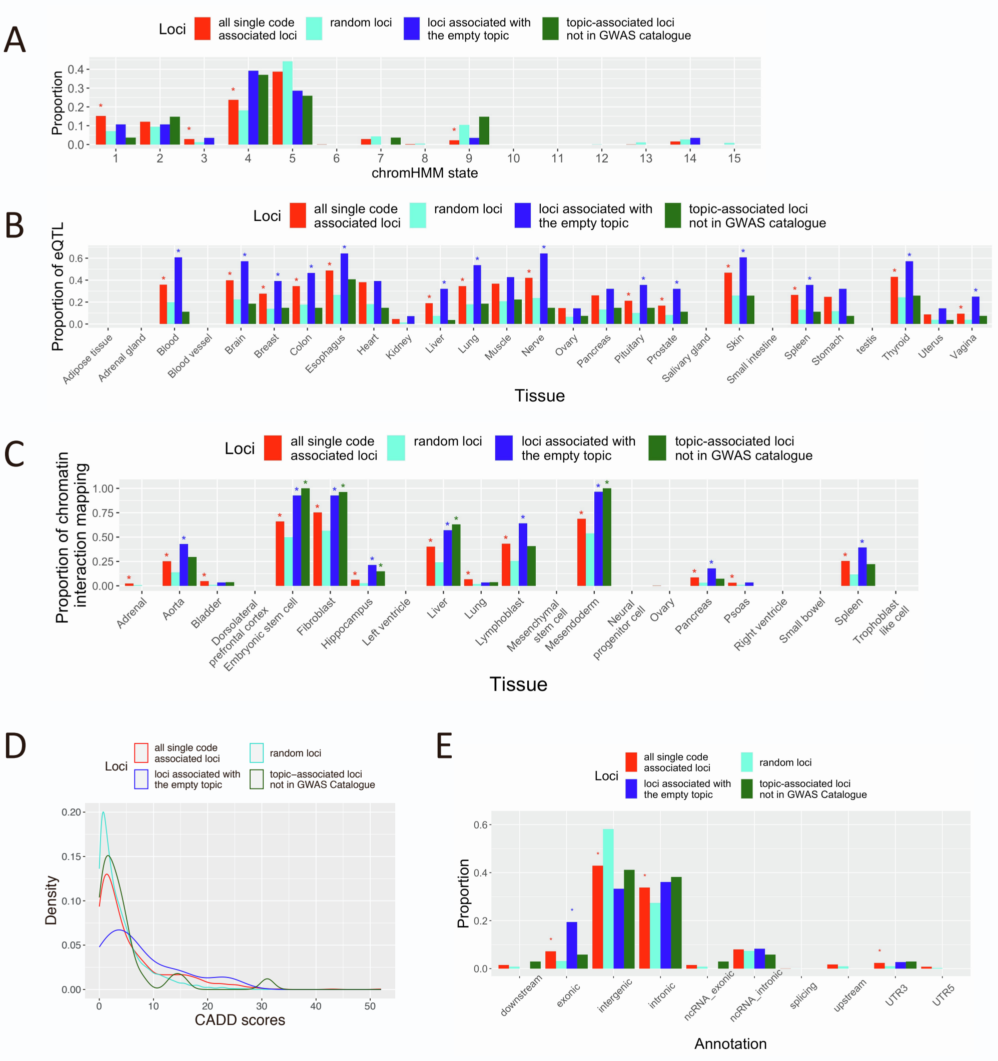

Supplemental Figure 12: **Validation of the empty topic associated loci and topic-associated loci not in the GWAS catalogue for the top-436 UKB dataset, related to Figure 6. A.)** Proportions of four groups of SNPs that are in different chromHMM states. The first group contains lead SNPs associated with at least one ICD-10 code (823); the second group contains 5,000 random SNPs (4,649 lead SNPs); The third group contains lead SNPs associated with the empty topic (28); The fourth group contains lead SNPs associated with at least one topic and have no record in the GWAS catalogue (27). The proportions of the other three groups are compared with the second group (random SNPs) respectively, and significant differences in proportions (two sided two-proportion Z-test, adjusted P-value<0.05, Bonferroni correction for the 15 chromHMM states) are marked with asterisks above the bars. The numeric results are in Table S7L. **B.)** Proportions of the four groups of SNPs in panel A being eQTL in different tissues. The numeric results are in Table S7L. **C.)** Proportions of the four groups of SNPs in panel A having chromatin interaction with genes in different tissues. The numeric results are in Table S7L. **D.)** Density plot for the CADD scores of the four groups of SNPs in panel A. **E.)** Proportions of the four groups of SNPs in panel A with different ANNOVAR annotations. The numeric results are in Table S7L.

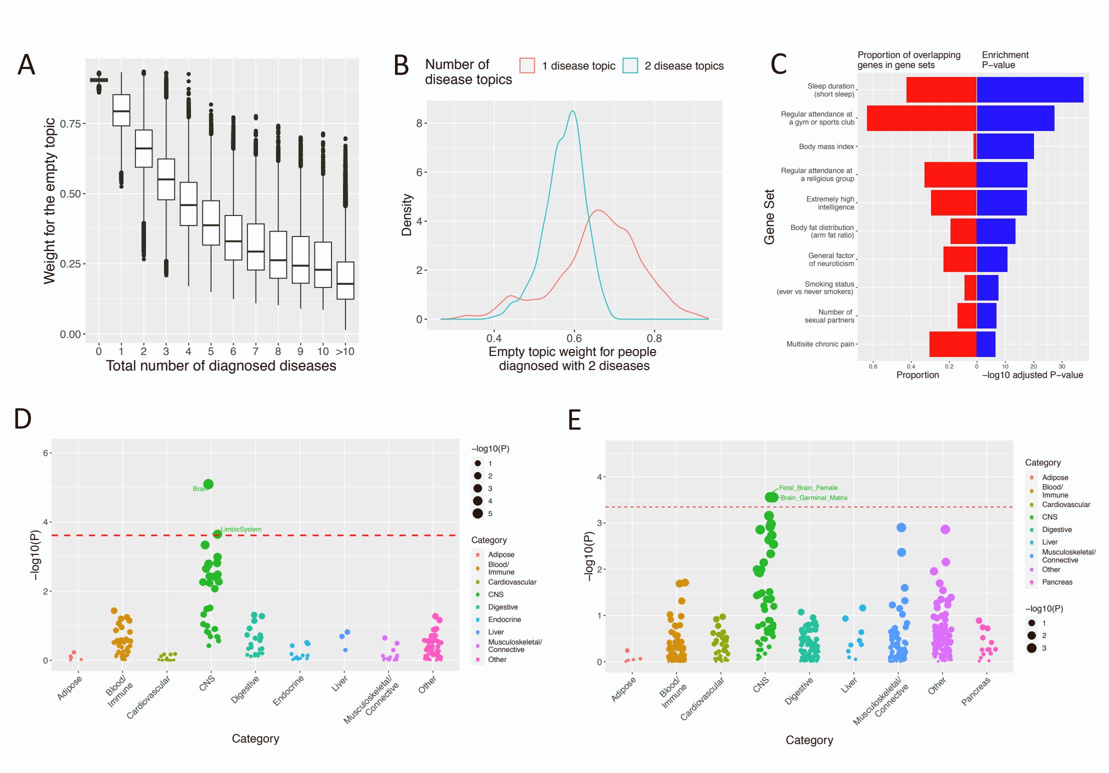

Supplemental Figure 13: Further analyses for the empty topic, related to Figure 6. A.) Weights for the empty topic for individuals diagnosed with different numbers of diseases in the top-436 UKB dataset. **B.)** Density plot for the empty topic's weight for individuals diagnosed with 2 diseases in the top-436 UKB dataset. These individuals were divided into 2 groups according to the number of disease topics generating their diseases. Individuals with large weights (>0.1) for 2 disease topics were assumed having 2 different topics generating the 2 diagnosed diseases. **C.)** Gene set enrichment analysis (GSEA) result for the empty topic. The empty topic associated genes are mapped from its associated SNPs based on physical distance on the genome. Reference gene sets used in the enrichment analysis are genes associated with different traits in the GWAS catalogue. The top-10 significantly enriched gene sets are shown. **D-E.)** Stratified LD score regression (sLDSC) for the topic-GWAS result of the empty topic on the top-436 UKB dataset using gene expression data (GTEx dataset) (panel D) and chromatin data (Roadmap Epigenomics dataset) (panel E) as annotations for cell types. Each dot in the plot corresponds to the P-value of enrichment for a specific cell type, and all cell types are divided into 9 groups according to their corresponding tissues. The numeric results are in Table S7N.
